# Granular by design: a fine-grained NER approach to context-aware medication extraction

**DOI:** 10.3389/frai.2026.1927272

**Published:** 2026-10-30

**Authors:** András Berzi, Ervin Berényi, Viktória Arató, Piroska Kallos-Balogh, Sándor Csaba Aranyi, Zita Képes, Miklós Emri

**Affiliations:** 1 Department of Nuclear Medicine and Translational Imaging, Institute of Medical Imaging, Faculty of Medicine, University of Debrecen, Debrecen, Hungary; 2 Doctoral School of Informatics, University of Debrecen, Debrecen, Hungary; 3 Department of Radiology and Imaging Science, Institute of Medical Imaging, Faculty of Medicine, University of Debrecen, Debrecen, Hungary; 4 Nuclear Medicine, Department of Medical Imaging, Diagnostic Units, University of Debrecen Clinical Centre, Debrecen, Hungary

**Keywords:** electronic health records, fine-grained entity recognition, Hungarian, medication context, medication extraction, named entity recognition, natural language processing

## Abstract

**Introduction:**

While structured electronic health records (EHRs) provide a baseline for medication history, they often fail to capture clinically important contextual information about medication use that is documented only in free-text clinical notes. A significant portion of critical clinical information – such as unreported adverse events or over-the-counter supplement usage – resides exclusively in unstructured free-text clinical notes. Our objective was to develop and evaluate a fine-grained context-aware Named Entity Recognition (NER) framework capable of transforming medication-related information from unstructured Hungarian clinical narratives into structured data suitable for clinical research.

**Methods:**

We developed a fine-grained context-aware Named Entity Recognition (NER) framework using the spaCy NLP library. Clinical documents were sourced from the UDBD-Health Research Data Repository of the University of Debrecen. A dedicated fine-grained annotation scheme was designed to categorize medication mentions into 4 contextual classes: Current Medication, Discontinued/No Medication, Adverse Events, and Allergies (CAVE). Documents were pre-annotated using rule-based vocabularies and manually refined in Label Studio. To evaluate model generalization and obtain a robust, leakage-free performance estimate, we employed 5-fold cross-validation with an inner validation split, in which model selection via early stopping used a validation subset drawn only from the training folds, while each outer held-out fold was reserved exclusively for final evaluation. To address the pronounced imbalance between medication context classes, we applied document-level oversampling of minority-class training examples. Model performance was assessed using Precision, Recall, and F_1_-score, reported as mean ± standard deviation across the five folds, and compared against a rule-based baseline built from the same vocabulary-matching procedure used for pre-annotation.

**Results:**

Two types of models were trained for medication information extraction. The fine-grained context-aware model (CME) achieved an overall F_1_-score of 0.944 ± 0.001 across five-fold cross-validation. At the entity level, CURRENT_MED yielded the highest F_1_-score (0.958 ± 0.001), followed by CAVE_MED (0.900 ± 0.023), NO_MED (0.820 ± 0.005), and AE_MED (0.689 ± 0.026), the latter exhibiting the greatest fold-to-fold variance. Applying document-level oversampling of minority-class examples (CME OS) resulted in an overall F_1_-score of 0.946 ± 0.002, with modest improvements in the minority-class F_1_-scores. The coarse-grained model (ME), trained on the single-entity MEDICATION recognition task, attained an F_1_-score of 0.968 ± 0.001 with minimal variance, substantially outperforming a rule-based vocabulary-matching baseline on the same task (F_1_ = 0.860 ± 0.003), which showed high Precision but comparatively low Recall. The models have been deployed into the institutional data pipeline, enabling automated structuring of medication documents from over 700,000 documents per year.

**Discussion:**

This study presents a fine-grained context-aware medication extraction system for Hungarian clinical documents. The developed models facilitate the transformation of unstructured medication narratives into structured medication data, providing a foundation for downstream pharmacological, epidemiological, and real-world evidence studies. Limitations include institutional data specificity, underrepresentation of adverse-event contexts, and document- rather than patient-level cross-validation splitting, and the lack of independent parallel annotation, which prevented calculation of inter-annotator agreement. Future work will focus on corpus expansion, inter-institutional validation, and the inclusion of additional attributes such as dosage and duration.

## 1 Introduction

Currently, researchers rely on approximately 20% of medical data that is structured, despite unstructured data – from electronic health records (EHR), administrative or clinical notes and other sources – comprising the vast majority (80%) (Nancy and Maheswari, 2020). This means, that a significant amount of data valuable for researchers is locked in unstructured formats, which is difficult and time-consuming to process using traditional methods, leading to its underutilization. Natural Language Processing (NLP) offers a solution by making this data more accessible and useable for clinical research.

Clinical reports contain a substantial amount of unstructured medication information, most of which remains inaccessible to large-scale clinical research and pharmacovigilance efforts. Free-text narratives describe medication names, active substances, drug classes, and even specialized dietary or magistral preparations, however this information is rarely captured in structured form. Systematical extraction of these details from narrative documents enables a deeper understanding of real-world drug utilization, adherence patterns, treatment histories, and adverse events.

The present study aims to establish a high-quality, context-aware annotation framework for extracting medication-related information from Hungarian clinical records. Rather than treating medication mentions as homogeneous entities – which can be misleading in clinical analyses – we differentiate them according to their clinical context – such as current use, discontinued or never-initiated therapy, adverse-event-related mentions, or documented allergies – thereby capturing distinctions that are crucial for downstream analytic and modeling tasks. To our knowledge, no context-aware medication annotation corpus and benchmark for Hungarian clinical narratives has been reported previously, motivating the development of the present dataset and evaluation framework. Ultimately, our goal is to create an annotated resource that can support the development and evaluation of NLP systems capable of reliable identification of medication exposure in unstructured Hungarian healthcare data.

Unstructured clinical narratives constitute one of the richest yet least accessible sources of information on medication exposure. Unlike structured medication lists, these documents contain subtle indicators of drug use, patient-reported symptoms, treatment failures, intolerance, allergies, adverse reactions, and terminated or unsuccessful therapies, as well as references to informal or off-label substance use. Harnessing these latent signals is fundamental for generating real-world evidence, understanding population-level medication usage, identifying adverse drug reactions that fall outside formal surveillance systems, and characterizing complex clinical profiles such as multimorbidity and polypharmacy. Such information also contributes to more accurate decision-support strategies, improved medication reconciliation, and the training of robust NLP tools tailored to medical text. Beyond pharmacovigilance applications, such structured medication information may also support clinical trial recruitment, retrospective cohort identification, and integration of routinely collected clinical data into research databases. A context-sensitive extraction approach is imperative, as the clinical implications of a medication that is currently taken differ substantially from those of a discontinued therapy or an agent associated with an adverse event – distinctions that cannot be captured by keyword-based methods alone.

The current state of the art in context-aware medication Named Entity Recognition (NER) is increasingly defined by Transformer-based models (Chen et al., 2023), which have demonstrated superior ability to capture nuanced clinical context compared to earlier architectures (Keraghel et al., 2024). For English clinical texts, models fine-tuned for medication extraction – such as those based on BioBERT (Lee et al., 2020), ClinicalBERT (Alsentzer et al., 2019) or Gatotron (Yang et al., 2022) – regularly achieve F_1_-scores above 0.90 for recognizing medication names and associated attributes like dosage and route in shared task evaluations (Henry et al., 2020). Although, performance on more granular, context-classification tasks – such as distinguishing between current, historical, adverse event-related, or allergic medication mentions – remains more variable, with leading systems in recent challenges like the n2c2 2022 shared task reporting F_1_-scores typically ranging from 0.80 to 0.92 (Mahajan et al., 2023). This highlights the persistent complexity of modeling clinical context, where subtle linguistic cues and document-level dependencies are critical.

Beyond English, recent work has begun to chart the multilingual frontier of medication NER, though progress is uneven across languages. For better-resourced European languages, Transformer-based approaches have demonstrated meaningful transferability, with studies investigating multilingual medication detection across German, French, Spanish, and English, exploring the extent to which existing annotated corpora and multilingual models can improve the situation in non-English clinical NLP settings (Raithel et al., 2025). Structured shared tasks have further accelerated multilingual benchmarking: the BioASQ 2024 MultiCardioNER challenge (Lima-López et al., 2024) evaluated medication recognition from cardiology clinical case reports in Spanish, English, and Italian. Top-performing BERT-based systems achieved F_1_-scores of 0.9209 for Spanish, 0.9174 for English, and 0.889 for Italian medication recognition, with results consistently showing that fine-tuned monolingual models outperformed both multilingual fine-tuning and zero-shot large language model prompting (Danu et al., 2025).

For lower-resource languages such as Hungarian (Jelencsik-Mátyus et al., 2023), the landscape is markedly sparser; while foundational language models like huBERT (Nemeskey, 2021; Nemeskey, 2020) provide a strong starting point, there are no widely reported, high-performance NER systems specifically tailored for medication extraction in Hungarian clinical narratives. Recent work has begun to benchmark conventional and deep learning-based approaches for general-purpose named entity recognition in unstructured Hungarian medical reports (Bogacsovics et al., 2024), but this line of work does not address medication-specific or context-aware extraction, underscoring a significant gap that the present study aims to address.

## 2 Materials and methods

The clinical documents were collected from the UDBD-Health Research Data Repository, which mainly consists of electronic health records from the University of Debrecen. We created vocabularies to pre-annotate the collected documents, which were manually corrected in Label Studio. Leveraging the spaCy NLP framework, we developed NER models to identify medication entities and their contextual attributes within clinical documents. These models were designed to process accurately and efficiently large-scale unstructured medical records, that can be used by researchers.

### 2.1 spaCy

SpaCy is an open-source Python library for advanced Natural Language Processing. It’s a production-oriented framework built upon current research, designed for robustness and performance in real-world applications. The spacy-transformers package integrates transformer models directly into spaCy’s pipeline as modular components, which allows the convenient use of state-of-the-art transformer architectures. It is compatible with all models from the HuggingFace (AI Community, n.d.) transformers library, including a Hungarian model, huBERT. The architecture of the medication extraction pipeline, from raw clinical text through tokenization, transformer encoding, and the fine-grained (CME) and coarse-grained (ME) NER models, is illustrated in Figure 1.

**Figure 1:**
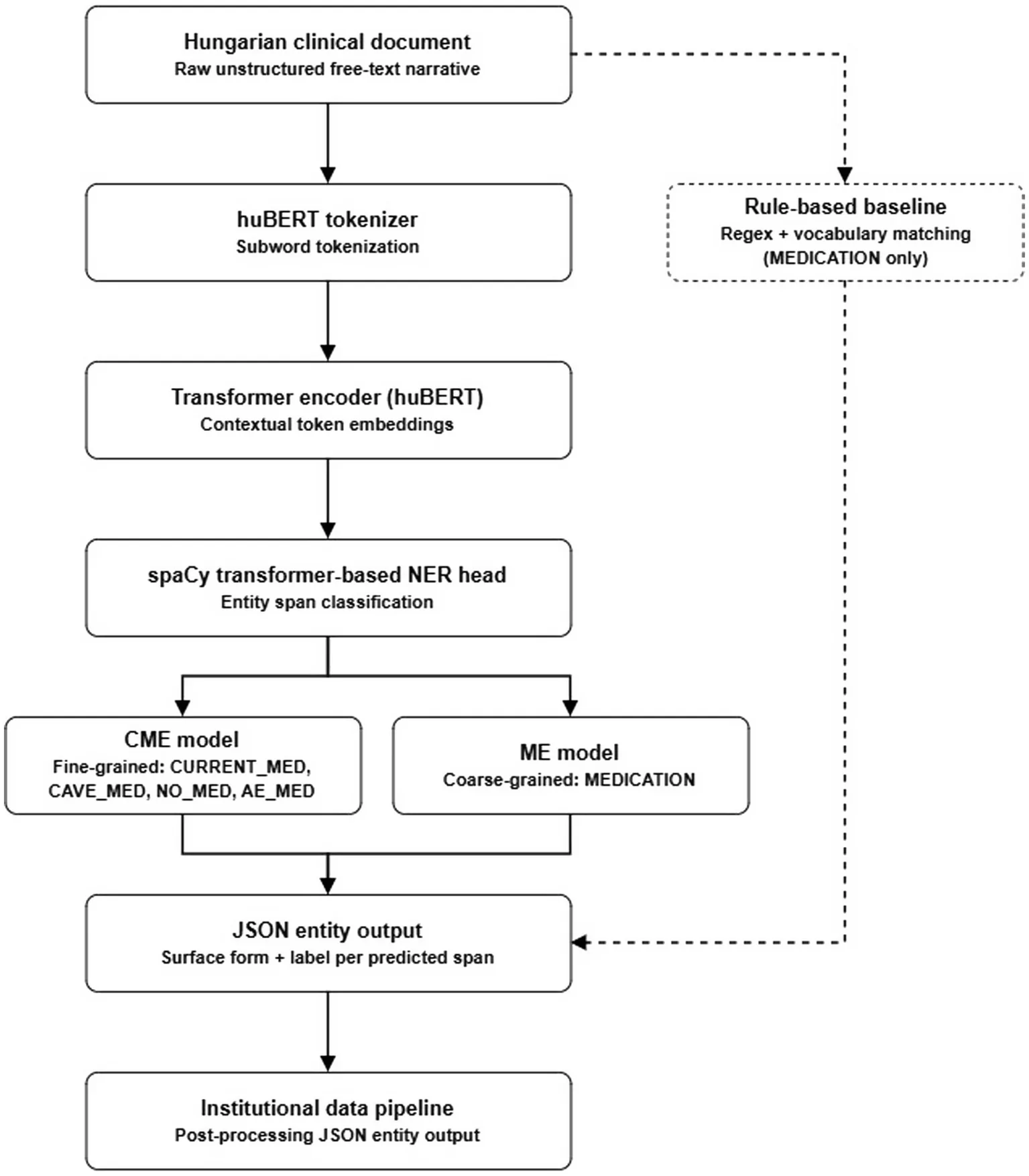
Model architecture diagram of the medication extraction pipeline.

### 2.2 Datasets

Due to the lack of available domain-specific Hungarian resources, we created our own manually annotated Hungarian clinical corpus. The data for this study was sourced from the UDBD-Health Research Data Repository, which contains over 57 million de-identified documents from various sources (Berzi et al., 2025). Due to its exclusively unstructured nature, our analysis focused on inpatient documents containing medications. For the corpus we collected 6,000 documents from the most common document types, including medical histories, status reports and discharge summaries. The corpus comprised 5,715 unique patients, with minimal recurrence of individual patients across documents, indicating that the corpus predominantly reflects distinct clinical cases rather than repeated documentation of the same encounters. The corpus contained 92,992 annotated medication entities and approximately 2.09 million tokens, corresponding to an average of 15.50 entities and 347.98 tokens per document.

### 2.3 Context differentiation

Mere entity recognition of drug names is insufficient for clinical decision support. Without contextual differentiation, a mention of a drug could erroneously imply current usage when, in reality, it refers to a known allergy or a discontinued therapy. To ensure that each medication mention carries clinically interpretable meaning, we assigned every instance to a well-defined contextual category. Our methodology emphasizes the categorization of drugs into four distinct contexts: Current Medication, Discontinued/No Medication, Adverse Events, and Allergies (Cave). Mentions describing medications currently taken by the patient, including historical and suggested medications, were distinguished from those referring to discontinued, previously used, or explicitly not taken drugs. Mentions linked to adverse events – including side effects, inefficacy, intolerance, or toxicity – were marked separately, while references to allergies or hypersensitivity were identified as a distinct subtype due to their clinical relevance. This contextual differentiation was intended not only to enhance the expressiveness of the dataset but also to enable downstream models to infer medication-related events rather than merely recognize medication names.

### 2.4 Tag selection and justification

Annotators were instructed to treat the following five types of textual expression as medication mentions, without distinguishing at the annotation level between specific drug names, active substances, and drug classes; all were treated as instances of the same underlying entity type. Explicit drug names, including official brand names together with any accompanying manufacturer designation (e.g., “Coverex AS Komb Forte,” “Vitamin D3 Fresenius,” “Bisoprolol Sandoz”), were annotated as part of a single span. Active substances (e.g., paracetamol, amoxicillin) and drug classes (e.g., antipyretic, anticoagulant, NSAID) were annotated identically whenever they appeared in place of, or alongside, a specific product name. Commonly used abbreviations referring to medications or active substances (e.g., CsA for cyclosporine A), including standardized abbreviations for infusion-protocol components used in oncological therapies, were also annotated as medications, according to a dedicated reference list. Dietary supplements and specialized medical-purpose food products available both within and outside pharmacies (e.g., magnesium, folic acid, Nutridrink) were likewise annotated as medications, reflecting their relevance to comprehensive medication-exposure assessment. Latin-named magistral (compounded) preparations following the Formulae Normales formulary, frequently appearing in abbreviated form (e.g., “Ung. Hydrophil. Non.”), were annotated as medications as well. Medications that have since been withdrawn from the market (e.g., Trexan) were annotated identically to currently marketed products, and misspelled or orthographically variant drug names (e.g., “i” substituted for “y” as in Memoril/Memoryl, or “c” substituted for “k” as in Betaloc/Betalok) were annotated as they appeared in the original text, without normalization, to preserve the heterogeneity of real-world clinical documentation.

Rather than annotating medications as a generic entity type and subsequently assigning context, annotators assigned each medication mention directly to one of four fine-grained, context-specific labels – CURRENT_MED, NO_MED, AE_MED, or CAVE_MED – in a single annotation pass. The CURRENT_MED label was applied to medications the patient is reported to be currently taking (e.g., “Medications: Concor, Adexor, Syncumar”); historical and recommended medications – including therapies proposed, planned, or advised by the treating physician but not yet confirmed as ongoing – were likewise labeled CURRENT_MED at the annotation stage, with the distinction between actively ongoing, historical, and recommended use left for downstream processing rather than resolved during annotation. The NO_MED label was applied to medications the patient previously took but has since discontinued, or medications explicitly reported as never having been taken (e.g., “The patient discontinued bisoprolol two months ago”). The AE_MED label was applied to medications associated with adverse events, including side effects, overdose, or therapeutic inefficacy (e.g., “Trental caused an adverse reaction,” “drug overdose,” “ineffective”), information considered particularly valuable for pharmacovigilance research. The CAVE_MED label was applied to medications to which the patient has a documented allergy (e.g., “Cave: lidocaine”).

### 2.5 Fine-grained NER

Fine-grained Named Entity Recognition extends traditional NER by classifying entities into more specific and detailed subcategories – distinguishing, for instance, between government agencies, educational institutions, and commercial companies rather than grouping them all under a single “organization” label. This deeper categorization demands that models develop stronger language understanding capabilities and handle complex challenges such as increasing entity categories, blurring entity boundaries, and fine-grained feature recognition (Yang et al., 2024).

A key challenge in advancing fine-grained NER is the construction of sufficiently rich and diverse taxonomies; MultiCoNER v2 (Fetahu et al., 2023) addresses this by introducing a fine-grained taxonomy of 33 entity sub-types across 6 coarse categories, incorporating realistic noise and multiple languages to better reflect the disambiguation challenges faced by practical NER systems. Fine-grained NER presents challenges such as distinguishing between subtypes that fall under the same coarse category. For example, correctly classifying fine-grained types of a MEDICATION entity – such as Current Medication, Discontinued/No Medication, Adverse Events, and Allergies – demands a strong contextual understanding and frequently relies on external medical knowledge. Since the same drug name can appear across multiple fine-grained categories depending on the clinical context (e.g., a drug mentioned as a current prescription in one record may appear as a reported allergy or a discontinued treatment in another), the surrounding context becomes essential for accurate type disambiguation. Our objective was to extract clinically meaningful medication mentions as self-contained entities that can be directly integrated into structured research databases. Encoding contextual information as fine-grained entity labels provides a simpler annotation scheme and enables a single-stage extraction workflow, whereas relation-based approaches require additional annotation and relation extraction steps that are beyond the scope of the present study.

### 2.6 Annotation scope and process

The annotation process was carried out by a team of three annotators, all of whom were clinical staff members with prior experience in medical annotation tasks. The lead annotator was a clinical pharmacist with more than 10 years of clinical experience and supervised the entire annotation workflow. Annotation followed a supervised two-stage procedure designed to produce a high-quality gold-standard corpus: in the first stage, medication mentions and their contextual labels were manually reviewed and annotated following automated pre-annotation; in the second stage, the lead annotator reviewed and, where necessary, corrected the first-stage annotations to ensure quality and harmonization across the corpus. As the second stage constituted expert supervision and correction of the initial annotations rather than an independent parallel annotation, a formal inter-annotator agreement metric was not calculated; instead, annotation quality was ensured through this structured review and correction process, with all final annotations validated by the lead annotator before inclusion in the training corpus. While this procedure was chosen to maximize the consistency and quality of the final gold-standard annotations, it does not provide a quantitative estimate of inter-annotator reproducibility, which represents a limitation of the present corpus. The flow diagram of the annotation process is shown in Figure 2.

**Figure 2:**
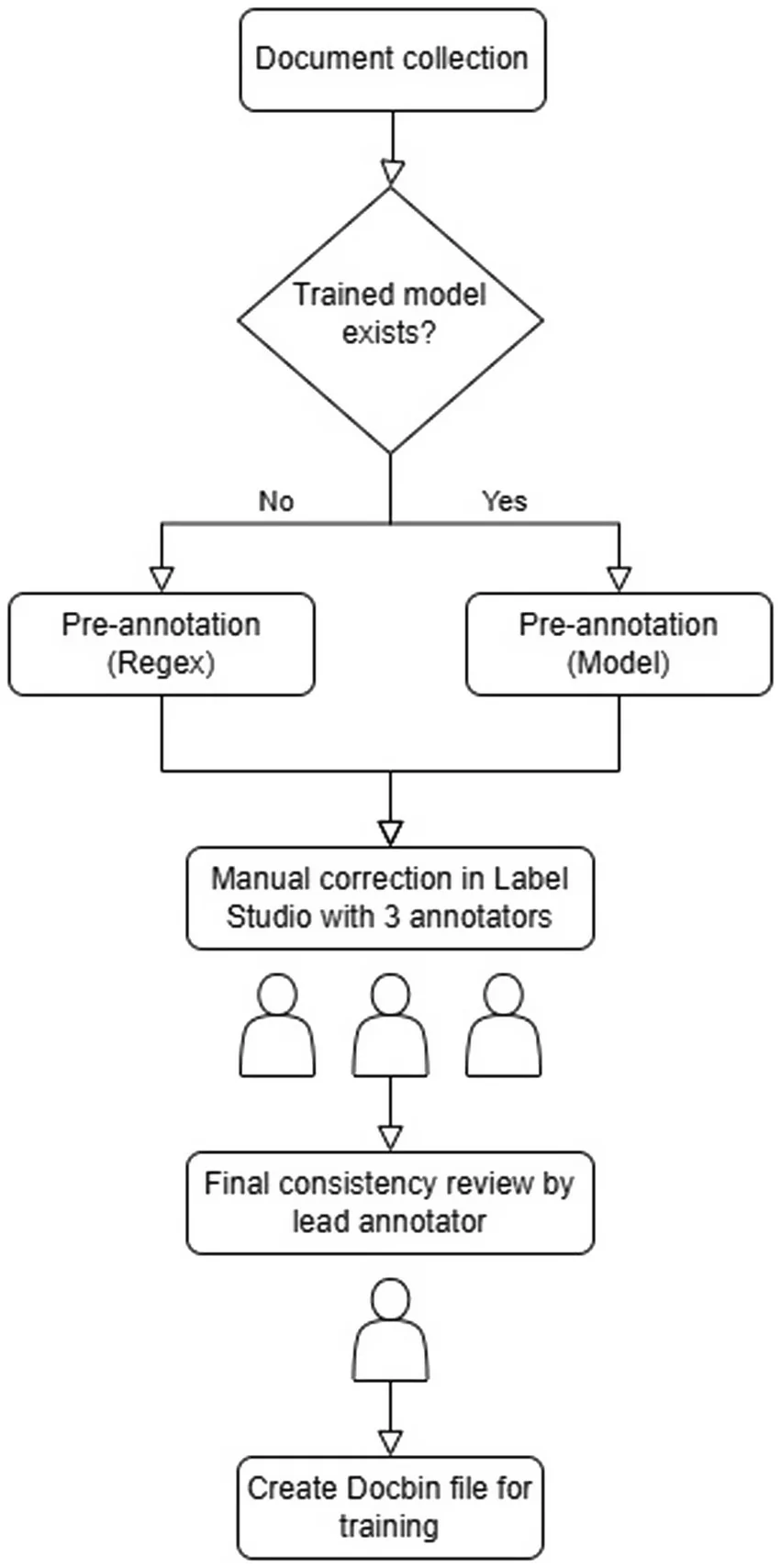
Flow diagram of the annotation process.

Within this study, Hungarian clinical reports were annotated with special attention to any expression referring to medications or pharmacological substances. This encompassed official drug names together with manufacturer information, active substances and drug classes, commonly used abbreviations, components of infusion protocols, dietary supplements and specialized nutrition products, magistral formulations, and even medications no longer commercially available. Misspellings and orthographic variants were intentionally preserved and annotated to reflect the heterogeneity of real clinical documentation.

We adopted a different strategy for building our training dataset compared with conventional medication extraction pipelines on medication extraction. Rather than first extracting the medication mention and then classifying it (Mahajan et al., 2023), we directly annotated medication mentions using fine-grained entity labels that simultaneously encode their context. Our labels were created to classify medications based on their contextual attributes, providing more nuanced information than medication names alone. The labels, their descriptions, and the number of labeled entities are provided in Table 1.

**Table 1:** Table of annotated entities.

Label name	Label description	Label count
CURRENT_MED	Medication the patient is currently taking, has taken in the past (historical), or has been advised to take (suggested). The specific temporal status is not distinguished at the annotation level.	83,990
CAVE_MED	Medication causing the patient sensitivity or allergy.	2,166
NO_MED	Medication the patient has stopped, never had to take, or has to stop taking.	4,735
AE_MED	Medication with adverse effect.	2,101

An automated pre-processing step using regular expressions was applied to the collected texts before the manual correction. A dedicated vocabulary was created for each context label, in addition to a separate vocabulary for medications. Manual correction of the pre-annotated documents was performed using Label Studio (Label Studio, n.d.), a flexible data labeling tool (Figure 3).

**Figure 3:**
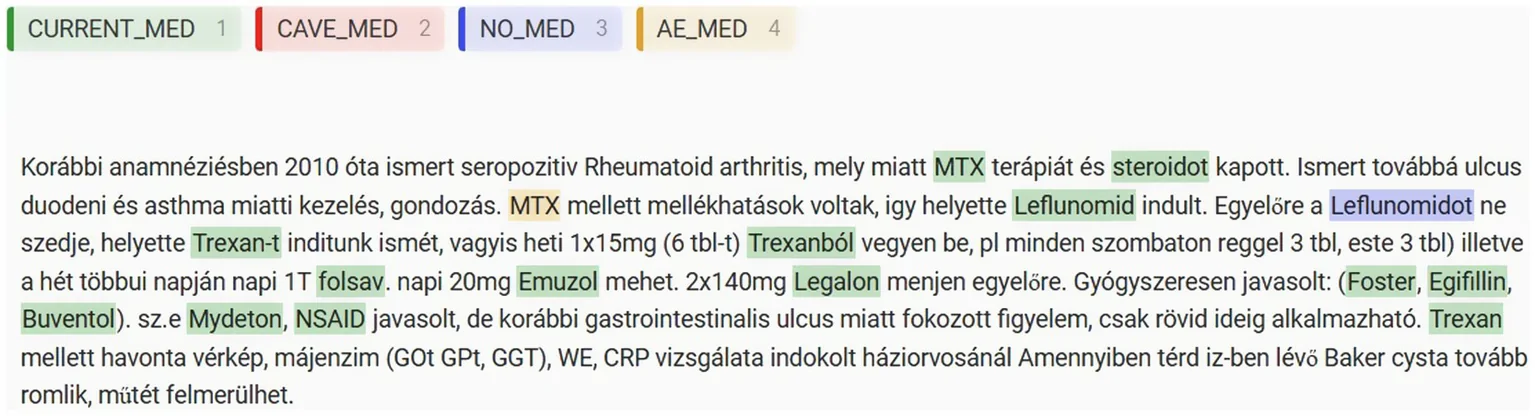
Sample annotation in label studio.

### 2.7 Training approach

Following the annotation phase, two NER models were developed to capture medication information at different levels of granularity, and the coarse-grained extraction task was additionally compared against a rule-based baseline. This baseline consisted of the same regular-expression and vocabulary-driven matching procedure used during pre-annotation, applied as a standalone extraction system on the basis of a dedicated medication vocabulary. Because lexicon lookup matches surface forms independently of their surrounding narrative, the baseline can only identify that a medication is mentioned, not the clinical context in which it occurs; it was therefore restricted to the single-entity MEDICATION task and no rule-based counterpart of the fine-grained task was feasible. The coarse-grained model (ME) addressed this same single-entity task, treating all medication mentions as a single undifferentiated entity type regardless of clinical context, and thus provides a direct comparison against the rule-based baseline. The fine-grained model (CME) was trained to extract context-aware medication mentions, distinguishing between four clinically meaningful entity types: CURRENT_MED, CAVE_MED, NO_MED, and AE_MED. All systems were evaluated on identical held-out data using the same exact span-matching criteria, enabling a direct comparison between rule-based and learned medication extraction as well as between coarse- and fine-grained entity schemes, and allowing us to assess both the added value and the additional complexity introduced by context-aware entity distinction.

To evaluate model generalization and obtain a robust performance estimate, we employed a 5-fold cross-validation with an inner validation split on the annotated corpus. The full dataset was partitioned into five mutually exclusive folds using random shuffling with a fixed random seed, ensuring reproducibility of the data splits across runs. Folds were split at the document level rather than the patient level; as a result, the same patient could appear in both the training and held-out test folds, though only through different encounters, since no document appeared in more than one fold. Given the minimal recurrence of individual patients across documents in the corpus, the practical impact of this document-level splitting on cross-fold data leakage is expected to be limited. For each outer fold, the remaining four partitions were combined and further split into an inner training set (90%) and an inner validation set (10%), the latter used exclusively for early stopping. The outer held-out fold was reserved entirely for final evaluation and was not used during training or model selection. A separate spaCy NER model was trained from scratch on each inner training set using the default Transformer-based pipeline, with early stopping based on the inner validation set’s F_1_-score to prevent overfitting.

To mitigate the pronounced class imbalance in entity frequency (Table 1), we implemented a custom weighted corpus reader that oversampled training documents containing minority entity classes. We registered a spaCy corpus reader (weighted_ner_corpus.v1) that loaded the annotated documents from each training partition, identified the fine-grained labels present in each document, and duplicated any document containing at least one rare label – CAVE_MED, NO_MED, or AE_MED – by a fixed oversampling factor before shuffling and yielding the resulting examples for training. This document-level oversampling increased the effective representation of minority-class contexts in each training epoch without altering the underlying held-out evaluation folds, and was applied only during CME model training on the inner training set within each outer fold, to avoid leaking duplicated examples into either the early-stopping or final evaluation partitions. The oversampling factor was set empirically (oversample_factor = 3) to balance improved minority-class Recall against the risk of overfitting on repeated document instances.

For training, a transformer-based spaCy NER pipeline built on the SZTAKI-HLT/hubert-base-cc Hungarian transformer, paired with spaCy’s TransitionBasedParser.v2 architecture, was used across all models. Long clinical documents were processed via strided spans (128-token windows with 96-token stride), mean-pooled and passed to the NER component through a TransformerListener; the upper projection layer was disabled, relying directly on the transformer’s contextual embeddings. Optimization used Adam with a linear warmup schedule, reaching a peak learning rate of 5e-5 after 250 warmup steps over 20,000 total training steps, with gradient accumulation of 3 to accommodate transformer memory demands. Padded batching (2,000 tokens per batch, oversized examples discarded) was used alongside a patience of 1,600 steps and evaluation every 200 steps, with entity-level F_1_ serving as the sole criterion for model selection during early stopping across all 5 folds. The detailed hyperparameters used for model training are shown in Table 2.

**Table 2:** Training components and hyperparameters.

Component	Architecture	Key hyperparameters
Transformer	spacy-transformers. TransformerModel.v3 (SZTAKI-HLT/hubert-base-cc)	window = 128, stride = 96, max_length = 512, mixed_precision = false
NER	spacy. TransitionBasedParser.v2	hidden_width = 64, maxout_pieces = 2, use_upper = false
Tok2vec Listener	spacy-transformers. TransformerListener.v1	pooling = mean, grad_factor = 1.0
Optimizer	Adam.v1	LR = 5e-5, warmup = 250 steps, grad_clip = 1.0, L2 = 0.01
Batcher	spacy.batch_by_padded.v1	size = 2000, buffer = 256, discard_oversize = true
Training	–	accumulate_gradient = 3, max_steps = 20,000, patience = 1,600, eval_frequency = 200
Scoring	–	ents_f = 1.0

Training was performed on a workstation equipped with an NVIDIA A100 PCIe GPU.

### 2.8 Evaluation metrics

The performance of the models was assessed with the conventional evaluation metrics for Named Entity Recognition: Precision, Recall, and F_1_-score. Precision quantifies the fraction of correctly identified entities among all predictions, whereas Recall quantifies the fraction of correctly identified entities from all ground truth annotations. A True Positive (TP) is a correctly identified entity, a False Positive (FP) is an incorrectly predicted entity, while False Negative (FN) is a missed ground-truth entity. Due to the individual limitations of Precision and Recall, the standard practice is to combine them using their weighted harmonic mean, the F-score. The F-score is evenly balanced between Precision and Recall when *β* = 1 (the F_1_-score). Values of β > 1 weight Recall more heavily, while β < 1 gives greater weight to Precision.


$$\text{Precision}=\frac{\mathrm{TP}}{\mathrm{TP}+\mathrm{FP}}$$

$$\text{Recall}=\frac{\mathrm{TP}}{\mathrm{TP}+\mathrm{FN}}$$

$$F-\text{score}=\frac{(\beta^{2}+1)*\text{Precision}*\text{Recall}}{\beta^{2}*\text{Precision}+\text{Recall}}$$

$$F_{1}-\text{score}=\frac{2*\text{Precision}*\text{Recall}}{\text{Precision}+\text{Recall}}$$

Final performance metrics – Precision, Recall, and F_1_-score – were computed on each outer held-out test fold, which was not used for training or checkpoint selection, using exact span matching, requiring both boundary and label agreement for a prediction to be counted as correct. The reported scores represent the mean and standard deviation across all five folds, providing an estimate of both model performance and variance attributable to training data composition.

## 3 Results

### 3.1 Cross-validation results

The CME model achieved an overall F_1_-score of 0.944 ± 0.001 across the five outer test folds, reflecting strong and stable performance with minimal fold-to-fold variance. At the entity level, CURRENT_MED was the best recognized category with an F_1_-score of 0.958 ± 0.001, followed by CAVE_MED (0.900 ± 0.023), NO_MED (0.820 ± 0.005), and AE_MED (0.689 ± 0.026), the latter exhibiting the greatest fold-to-fold variance among the four entity types. Applying document-level oversampling of minority-class training examples (CME OS) yielded an overall F_1_-score of 0.946 ± 0.002, with CURRENT_MED at 0.959 ± 0.002, CAVE_MED at 0.905 ± 0.017, NO_MED at 0.830 ± 0.009, and AE_MED at 0.694 ± 0.025. Across both CME variants, AE_MED consistently showed the lowest F_1_-score and the highest variance, while CURRENT_MED remained the most reliably recognized category, reflecting its high frequency and relatively unambiguous contextual cues in the training data. The results are summarized in Table 3, and the corresponding normalized results are displayed in Figure 4. Values in Figure 4 represent row-normalized proportions and are rounded to two decimal places; consequently, values displayed as 0.00 may represent either zero or very small proportions.

**Table 3:** Cross-validation results.

Model	Entity type	Precision	Recall	F_1_
CME	Overall	0.943 ± 0.003	0.945 ± 0.002	0.944 ± 0.001
	CURRENT_MED	0.957 ± 0.004	0.960 ± 0.003	0.958 ± 0.001
	CAVE_MED	0.888 ± 0.022	0.913 ± 0.025	0.900 ± 0.023
	NO_MED	0.833 ± 0.037	0.810 ± 0.026	0.820 ± 0.005
	AE_MED	0.702 ± 0.053	0.681 ± 0.040	0.689 ± 0.026
CME OS	Overall	0.944 ± 0.004	0.947 ± 0.005	0.946 ± 0.002
	CURRENT_MED	0.958 ± 0.004	0.961 ± 0.005	0.959 ± 0.002
	CAVE_MED	0.890 ± 0.026	0.921 ± 0.023	0.905 ± 0.017
	NO_MED	0.835 ± 0.018	0.825 ± 0.029	0.830 ± 0.009
	AE_MED	0.715 ± 0.048	0.675 ± 0.011	0.694 ± 0.025
Baseline (REGEX)	MEDICATION	0.984 ± 0.001	0.764 ± 0.004	0.860 ± 0.003
ME	MEDICATION	0.969 ± 0.001	0.966 ± 0.002	0.968 ± 0.001

**Figure 4:**
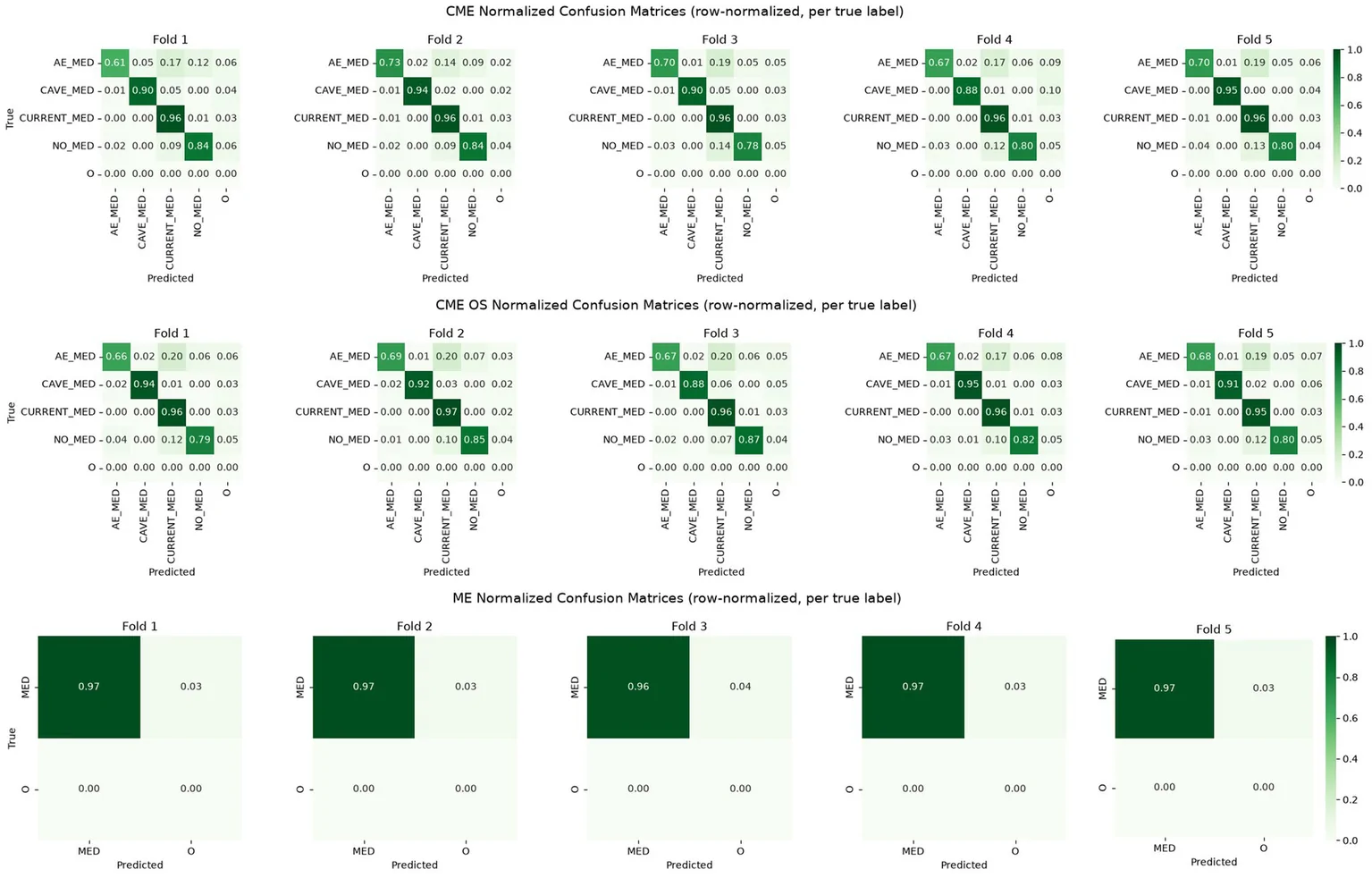
Normalized confusion matrices (row-normalized, per true label).

The rule-based baseline, applied to the single-entity MEDICATION task using the same regular-expression and vocabulary-matching procedure used for pre-annotation, was evaluated on the same 5 outer test folds used for the ME models, achieving an F_1_-score of 0.860 ± 0.003, with high Precision (0.984 ± 0.001) but comparatively low Recall (0.764 ± 0.004). This Precision-Recall profile reflects the nature of lexicon-based matching: exact vocabulary hits are reliably correct, but the fixed vocabulary cannot capture morphological variation, misspellings, or medication mentions not covered by the predefined lists. The ME model, trained on the same coarse-grained MEDICATION task, achieved an F_1_-score of 0.968 ± 0.001, with balanced Precision (0.969 ± 0.001) and Recall (0.966 ± 0.002) and minimal variance across folds, indicating stable performance regardless of fold composition.

### 3.2 Application

To assess inference speed, we ran evaluations on the same hardware configurations that were utilized in the training phase. Comparing inference speed across our models, both CME and ME models performed at approximately the same rate, averaging around 19,500 words per second. This consistency suggests that the architectural similarities between the two types of models result in comparable computational efficiency at inference time, with neither model presenting a meaningful speed advantage over the other.

To support the final application, we processed our documents on a year-by-year basis, with each annual batch comprising approximately 700,000 to 800,000 documents. This incremental approach allowed us to maintain computational efficiency and ensure data consistency across temporal segments. Processing a single year’s dataset required roughly three and a half hours to complete, enabling structured analysis while keeping memory and processing demands manageable. This method not only facilitated smoother pipeline execution but also enabled easier tracking of yearly trends and validation of intermediate results before proceeding to the next batch.

The medication extraction pipeline produces a structured output where each clinical text block is annotated with named entities serialized as a JSON array. Each element in the array is an object containing a surface form field and an entity class label field, capturing both what was extracted and how it was classified. To make this nested structure analytically tractable, the JSON array is first deserialized against a predefined schema, then exploded so that each individual entity occupies its own flat row. This transformation converts the one-row-per-document format into a one-row-per-entity format, preserving patient and encounter identifiers as foreign keys across every expanded row. The resulting tabular structure is immediately joinable with structured clinical tables and well-suited for aggregation, deduplication, and frequency analysis at both the encounter and patient level.

## 4 Discussion

The presented models can efficiently identify and categorize medication-related entities within Hungarian clinical documents from the University of Debrecen, achieving high performance for the most frequent contextual categories. Leveraging spaCy’s production-oriented design, the models have been successfully deployed and are currently integrated into our existing data processing pipeline, where they structure unstructured medication narratives for downstream analysis. A notable limitation is the institutional specificity of our training data, which may affect model performance in external clinical settings. This constraint lies beyond the current study’s scope but highlights an opportunity for future inter-institutional collaboration within the UDBD-Health framework. Another consideration is corpus composition: by focusing on common inpatient document types, certain contextual categories – particularly adverse event contexts – were underrepresented, as evidenced by lower F_1_-scores for AE_MED. Incorporating a wider variety of clinical note types could mitigate this imbalance and improve model generalizability across medication-related contexts. In addition to its lower frequency, adverse-event-related medication mentions typically depend on broader clinical context and more heterogeneous linguistic expressions, making them intrinsically more challenging to recognize than explicit current medication mentions. This challenge is consistent with a systematic comparative analysis of large pretrained language models on contextualized medication event extraction, which confirms that reliable recognition of contextual medication events remains an area of active methodological development even for well-resourced languages (Abdul-Quddoos et al., 2025), reinforcing the relevance of continued benchmarking efforts such as the present study for languages, including Hungarian, that remain underrepresented in this literature.

A further limitation concerns the cross-validation splitting strategy: folds were constructed at the document level rather than the patient level, meaning that the same patient could appear in both the training and held-out test folds, although only through different encounters, since no individual document appeared in more than one fold. Given the minimal recurrence of individual patients across documents in the corpus (5,715 unique patients across 6,000 documents), the practical impact of this document-level splitting on cross-fold data leakage is expected to be limited. Nonetheless, patient-level fold splitting would provide a stricter guarantee against any residual leakage and is recommended for future iterations of this work.

Similarly, a limitation concerns the annotation procedure itself: because the second annotation stage constituted expert review and correction of the first-stage annotations rather than an independent parallel annotation, a conventional inter-annotator agreement metric could not be calculated. While the structured two-stage review and lead-annotator validation were designed to maximize the consistency and quality of the final gold-standard corpus, this workflow does not provide a quantitative estimate of inter-annotator reproducibility, and the reported annotation quality therefore rests on the review process itself rather than on a statistically verifiable agreement score. Future annotation efforts could incorporate a double-annotation subset, evaluated independently before adjudication, to enable formal reproducibility assessment alongside the current quality-control approach.

Most recognition errors occurred in the AE_MED and NO_MED categories, where contextual interpretation depended on longer narrative spans, implicit negation, or heterogeneous clinical wording. In contrast, CURRENT_MED mentions generally appeared in more standardized prescription-like contexts, contributing to their higher recognition accuracy.

The developed fine-grained context-aware NER models not only have potential for pharmacological data processing but also hold great promise for application in clinical research. Such methods may facilitate downstream analyses of medication exposure and medication-related clinical information, supporting pharmacological and epidemiological research. Automated extraction enables large collections of routine clinical documentation to be integrated more efficiently into research databases, complementing structured clinical data for observational and real-world evidence studies. In addition, automated and rapid extraction of medication-related information from clinical documentation can decrease the need for time-consuming manual data processing and minimize the risk of human error, thereby enabling more precise and scalable data management in research settings. Structured medication information derived from clinical narratives may also facilitate retrospective cohort identification and support the selection of patient populations for clinical studies. Overall, by improving data quality and supporting data-driven decision making, such approaches may contribute to more efficient clinical research.

## 5 Conclusion

In this study, we created a novel Hungarian corpus focused on medication extraction from Hungarian EHR documents. Using this dataset, we developed two types of NER models to identify and extract drug names with contextual differentiation. The resulting models facilitate structured medication data retrieval from unstructured EHR documents, enabling compliant reuse for clinical research.

Looking ahead, future work will focus on corpus expansion through the inclusion of additional document types and data from other Hungarian healthcare institutions, which will support inter-institutional validation and improve model generalization. Furthermore, we plan to extend the annotation schema to capture finer-grained attributes such as dosage, frequency and duration enabling even more detailed pharmacovigilance and real-world evidence studies. Continued collaboration within the UDBD-Health ecosystem and the adoption of emerging Transformer-based language models for Hungarian will further enhance the robustness and applicability of the system across diverse clinical settings.

## Data Availability

The datasets presented in this article are not readily available because the datasets presented in this article are not available due to patient confidentiality and privacy regulations. Requests to access the datasets should be directed to berzi.andras@med.unideb.hu.
